# Furin inhibits HSCs activation and ameliorates liver fibrosis by regulating PTEN‐L/PINK1/parkin mediated mitophagy in mouse

**DOI:** 10.1096/fba.2024-00221

**Published:** 2025-03-28

**Authors:** Yan‐Wei Song, Yu‐Hua Zhu, Ming‐Ze Ma

**Affiliations:** ^1^ Department of Infectious Diseases Shandong Provincial Hospital Affiliated to Shandong First Medical University Jinan China

**Keywords:** Furin, hepatic stellate cells, liver fibrosis, mitophagy, PTEN‐long

## Abstract

Hepatic Stellate cells (HSCs) play an important role during liver fibrosis progression; more and more evidence indicates that mitophagy greatly regulates HSCs activation. HSCs mitophagy mainly depends on the classical PINK1/Parkin pathway, which can be strongly regulated by phosphatase PTEN‐long (PTEN‐L). PTEN‐L can be cleaved by Furin that leading to functional changes in the tumor regulation process. However, the impact of the interaction between Furin and PTEN‐L on HSCs mitophagy remains unclear. Therefore, this study aims to explore the role of Furin in HSCs activation and liver fibrosis and its potential mechanisms. Our results revealed that Furin expression was obviously up‐regulated during HSCs activation and mice liver fibrogenesis. We also found that the activation of primary HSCs can be inhibited by Furin treatment in vitro. Besides, functional studies showed that LX‐2 cell proliferation and migration were obviously inhibited by Furin treatment. Further studies showed that mitochondrial membrane potential (MMP) was significantly reduced by Furin treatment, and the knockdown of PTEN‐L expression caused similar effects. These results demonstrated the role of Furin in promoting HSCs mitophagy but leading to inhibition of HSCs persistent activation. Furthermore, we constructed a liver fibrosis mouse model by CCl4‐induced method and found that forced expression of Furin caused alleviation of liver fibrosis in CCl4‐induced mice. Our findings provide a new clue for understanding liver fibrogenesis and highlight the therapeutic potential of Furin for hepatic fibrosis.

## Graphical abstract

Furin interacted with PTEN‐L and activated PINK1/Parkin‐mediated mitophagy in HSCs. Furin inhibited HSC activation and alleviated liver fibrosis by regulating the PTEN‐L/PINK1/Parkin axis.

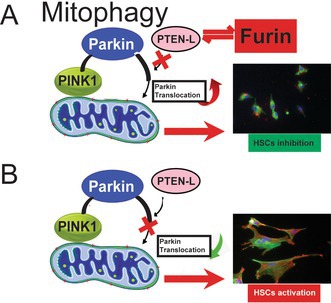

## 1 INTRODUCTION

Liver cirrhosis is a common chronic disease which is characterized by the hypertrophy of fibrous tissue and the formation of pseudolobuli in the liver.^1^ Liver fibrosis can be considered an early stage of liver cirrhosis which might be partly reversed by proper treatment.^2^ Chronic recurrent hepatitis usually leads to destruction of liver tissue structure.^2^ Therefore, it is urgent to discover new molecular targets for blocking liver fibrosis initiation and progression.

Hepatic stellate cells (HSCs) are widely considered as the crucial role during liver fibrosis initiation and progression.^3^ Blockage of HSCs persistent activation is an available and effective approach for liver fibrosis treatment.^4^ HSCs activation can be regulated by mitophagy, which has been widely studied in tumor and non‐tumor fields in recent years.^5^ The mitophagy process is mainly mediated by the PINK1/Parkin signaling pathway, and PTEN‐L is identified as an important inhibitor of PINK1/Parkin in mediating the mitophagy pathway.^6^, ^7^ However, the previous studies mainly focused on the inhibitory effect of PTEN‐L in tumor cell mitophagy regulation. The effect of PTEN‐L in regulating HSCs mitophagy remains unclear.

Therefore, we strive to explore new molecular mechanisms of PTEN‐L regulation for the sake of revealing a new target for liver fibrosis treatment.

Previous studies showed that Furin can extracellularly cleave PTEN‐L and generate a C‐terminal fragment with a tumor‐suppressive role.^8^ These important studies indicated that PTEN‐L can be identified as the substrate of the Furin enzyme, which causes its structural and functional changes. Therefore, we focus on exploring how Furin regulates HSC mitophagy through the PTEN‐L/PINK1/Parkin pathway.

In this study, we found the inhibitory effect of Furin in HSCs activation and even liver fibrosis. Furin was up‐regulated during HSCs activation and positively regulated mitophagy. The inhibitory effect of PTEN‐L on PINK1/Parkin mediated mitophagy was partly reversed by Furin. We also found that Furin positively regulated mitophagy in HSCs was PTEN‐L dependent. These results highlight Furin as a potential target to ameliorate or even reverse liver fibrosis.

## 2 MATERIALS AND METHODS

### 2.1 Clinical samples

All human liver tissues involved in this study were provided by the Department of Hepatobiliary Surgery and Organ Transplantation Surgery, Shandong Provincial Hospital affiliated with Shandong First Medical University. All the liver tissues were obtained with informed consent, and this study was approved by the Ethical Committee of Shandong Provincial Hospital affiliated with Shandong First Medical University (grant No. NSFC: No. 2020‐127).

### 2.2 Isolation of primary mouse HSCs

Wild type C57 mice were used for primary HSCs isolation, and animal studies were approved by the Ethics Committee of Shandong Provincial Hospital affiliated to Shandong First Medical University. The HSCs isolation involved in our study was carried out through the Nycodenz density gradient separation method. The isolated primary HSCs were cultured in low glucose Dulbecco's modified Eagle medium (DMEM) (Gibco, USA) supplemented with 20% fetal bovine serum (FBS). The isolated HSCs were cultured at 37°C and 5% CO_2_ condition.

LX‐2 cell (Immortalized human HSCs, Procell, Wuhan, China) was used for functional studies and cultured in high glucose DMEM medium supplemented with 10% FBS.

### 2.3 Immunohistochemistry stain

Liver specimens obtained from mice or humans were fixed with 4% paraformaldehyde, embedded in paraffin. The tissue sections were deparaffinized with xylene, hydrated with gradient alcohol, and used for IHC experiments. For immunohistochemistry (IHC) staining, the liver tissue sections were incubated in 3% hydrogen peroxide for 30 min in order to remove endogenous peroxidase activity. Then the sections were blocked with 5% BSA at room temperature for 1 h. Afterwards, the sections were incubated with primary antibodies at 4°C overnight. The primary antibodies used in this study included: anti‐α‐SMA (Sigma Aldrich A2547), anti‐Furin (Abcam, ab3467). The sections were then incubated with HRP‐coupled secondary antibodies at room temperature for 1 h. HRP‐DAB substrate chromogenic reagent was used in this IHC staining experiment, and the chromogenic reaction was controlled under an optical microscope. All the stained sections were dehydrated with gradient ethanol and sealed with neutral balsam. The IHC stain images were analyzed by two pathologists, respectively.

### 2.4 Sirius red and Masson Tri‐chrome stain

Liver tissue sections were dewaxed by xylene and gradient ethanol before the Sirius red stain experiment. The sections were then immersed in Sirius red solution (Picro Sirius Red Stain Kit, Abcam, ab150681) and stained for 20 min at room temperature. Afterwards, all the sections were dehydrated with gradient ethanol and sealed with neutral balsam at the end.

For Masson Tri‐chrome stain (Beyotime, C0189S), sections were firstly deparaffinized according to IHC steps. Then the sections were stained with Weigert hematoxylin solution for 5 min at room temperature and immersed in 1% hydrochloric ethanol for differentiation. Following steps included Ponceau staining for 10 min and washing with phosphomolybdic acid solution. Aniline blue stain was the most important step for collagen staining, and it can be terminated by running water flow. All the sections were dehydrated using gradient ethanol and sealed with neutral balsam finally.

### 2.5 Quantative real‐time PCR

The total RNA isolation was carried out using TRIzol reagent according to the manufacturer's instructions step by step (Takara, T9108, RNAiso Plus). Reverse transcription reaction was performed by using the reverse transcription kit for cDNA synthesis (Takara, RR047A). We chose the SYBR Green Real‐time PCR Master mixes reagent (Takara, RR820A) for PCR reaction and the CT (cycles to threshold) values were recorded. The results were presented as 2^−ΔΔCt^ values and analyzed by built‐in software from ABI 7500 PCR device.

### 2.6 Western blot

The protein extraction was carried out by using lysis buffer which contained 50 mMTris‐HCl, 150 mM NaCl, 1% Triton‐X 100, and 1 mM PMSF. Protein concentration was detected with the BCA method and mixed with 1 × loading buffer (contain SDS, β‐mercaptoethanol, et al.) before the blotting assay. Proteins were separated by SDS‐PAGE electrophoresis under constant voltage conditions. The nitrocellulose (NC) membrane was used for protein transfer, and this process was carried out under constant current conditions for about 1 h. Subsequently, the NC membrane was washed with TBST solution and blocked with 5% bovine serum albumin (BSA) solution at room temperature for 1 h.

#### 2.6.1 The primary antibodies incubation

The NC membrane was further incubated with the Alexa Fluor 680‐conjugated anti‐mouse or anti‐rabbit secondary antibodies (Life Technologies) avoiding light at room temperature for 1 h. All the fluorescence signals were captured and recorded by the Odyssey Imaging System (LI‐COR).

### 2.7 Lentivirus plasmid construction and cell transfection

HEK‐293T cells in the logarithmic phase were used for lentivirus packaging. We chose the pCDH‐CMV‐MCS‐EF1‐CopGFP‐T2A‐Puro vector as the cloning vector. The sequence of the Furin gene (NM_002569.4, 2385 bp) was obtained from the NCBI database for synthesizing its CDS sequence as the template for high‐fidelity PCR. The PCR amplification products were purified by using a gel extraction kit and digested by restriction endonuclease. Then, we performed the ligation assay by inserting the Furin CDS into the cloning vector with T4 DNA ligase. The recombinant vectors combined with PACK vectors (pPACKH1‐GAG, pPACKH1‐REV, pPACKH1‐VSV) were used for 293T cell transfection.

We then seeded 293T cells into 15 cm^2^ culture dishes at a density of 5 × 10^5^ cells/mL and cultured the cells in bovine serum‐free medium for 2 h before cell transfection. Cell transfection was carried out by Lipofectamine 2000 when cell density reached 70%–80%. We mixed PACK vectors, Furin recombinant vectors, Lipofectamine, Polybrene, and bovine serum‐free medium at room temperature for 15 min. We added the mixture into 293T cells drop by drop and cultured the cells at 37°C and 5% CO_2_. Finally, we collected the cell supernatant, which contained viral particles after vectors mixture transfection for 48 h, and filtered the virus solution by using the 0.45 μm filter membrane.

We prepared LX‐2 cells for lentivirus transfection. The LX‐2 cells were seeded into 10 cm^2^ culture dishes and washed with PBS and added Furin lentivirus solution drop by drop. After lentivirus transfection for 24 h, the cell supernatant was discarded, and the fresh DMEM solution was added. Puromycin was added for cell screening 72 h after lentivirus transfection. Owing to the Puromycin‐resistance gene included in the lentivirus vector, the living cells cultured in Puromycin medium were considered Furin‐lentivirus transfected cells.

### 2.8 Experimental animal model

C57BL/6J mice (6 weeks, 20–22 g weight) were purchased from the Experimental Animal Center of Shandong University. These animal experiments were approved by the Medical Ethics Committee of Shandong Provincial Hospital affiliated with Shandong First Medical University.

We carried out a CCl4‐induced experiment for liver fibrosis mouse model construction. The C57BL/6J mice in the model group (CCl4‐induced) were subcutaneously injected with 0.1 mL CCl4 /olive oil (1:3, v/v, dilation ratio) per 100 g (body weight) twice a week for 8 weeks. The C57BL/6J mice in the control group were subcutaneously injected with 0.1 mL olive oil only per 100 g (body weight) twice a week. For the empty vector group, mice were treated with the control lenti‐vector via tail vein injection twice a week in parallel with CCl4 injection. For the Furin OV (overexpression) group, mice were treated with the Furin lentivirus vector via tail vein injection twice a week in parallel with CCl4 injection in order to achieve Furin overexpression in vivo. Mice were sacrificed at 6 weeks after the first injection, and the livers were collected for further analysis.

### 2.9 Cell and tissue immunofluorescence staining

The primary HSCs and LX‐2 cells were used for cell immunofluorescence staining. The primary HSCs were isolated from mice livers by using a density‐gradient separation method and seeded into circular slides until attached growth. Then the slides were incubated with anti‐α‐SMA antibody (Sigma‐Aldrich) and Furin antibody (Abcam, Anti‐Furin antibody (ab3467)) for 75mins at room temperature. We then treated the slides with the Alexa Fluor conjugated secondary antibody at room temperature for 75 mins. Cell nuclei were stained with DAPI at room temperature for 30 mins.

For mitophagy detection, LX‐2 cells were divided into NC + TGF‐β group, Furin + TGF‐βgroup. LX‐2 cells in different groups were performed with immunofluorescence staining following the above steps and incubated with anti‐Tom20 antibody (Abcam, Anti‐Tom20 antibody (ab186735), Mitochondrial marker) and OPTN antibody (Abcam, Anti‐Optineurin antibody (ab313418)) for 75mins at room temperature. We then treated slides with the Alexa Fluor conjugated secondary antibodies at room temperature for 75 min.

Mice liver tissues from different groups were processed using tissue Immunofluorescence staining mainly for mitophagy evaluation. Liver tissue slides were dewaxed and hydrated, followed by antigen blocking with 5% bovine serum albumin. The tissue slides were incubated with anti‐HSP60 antibody (Abcam, Anti‐HSP60 antibody (ab59457)) and OPTN antibody (Abcam, Anti‐Optineurin antibody (ab313418)) for 75mins at room temperature. Finally, all slides were sealed with an anti‐quenching sealing agent. The images were observed and recorded using the fluorescence microscope (Carl Zeiss).

### 2.10 Immunoprecipitation assay

Cell lysates were prepared by using IP cell lysate (Pierce™ IP lysate buffer, ThermoFisher Scientific). Cell lysate was added to Protein A/G agarose beads and shook gently at 4°C for 10 min. Protein A/G agarose beads were removed by centrifugation, and Furin/PTEN‐L antibodies were added to the cell lysate. Centrifugal and elution steps were carried out following the IP kit manufacturer's instructions. The denaturation step was performed after the elution by using the boiling method. The IP products were identified using a western blot assay, which was already detailedly described in this article.

### 2.11 Flow cytometry for MMP detection

LX‐2 cells were divided into Furin + TGF‐βgroup, NC + TGF‐βgroup, et al. JC‐1 mitochondrial membrane potential Assay kit (Abcam, ab288313) was used for MMP detection following the manufacturer's instructions. LX2 cells in different treatment groups were digested and resuspended using pancreatin. Cell suspension was washed with pre‐cooling PBS buffer three times. Then the cell supernatant was abandoned and cell precipitation was added to JC‐1 dyeing solution (1 mL per tube) and incubated at 37°C for 20 min. During the JC‐1 dyeing process, JC‐1 dyeing buffer (5×) was diluted to a 1× working concentration with distilled water and then put in ice. The cell/JC‐1 dyeing mixture was washed with pre‐cooling 1× buffer twice before Flow cytometry detection.

### 2.12 ELISA assay

We carried out the ELISA assay in this study for the detection of COX and ATP concentration in cell supernatant. The human COX ELISA kit (FANKEW, Cat. No, F10842‐B) and the human ATP ELISA kit (FANKEW, Cat. No, F1967‐B) were used in this study for the detection of the target molecule. The sample package was carried out according to the manufacturer's instructions. Cell supernatant was diluted five times before being added into reaction pores and incubated at 37°C for 1 h. Every reaction pore was added 0.1 mL enzyme‐labeled antibody and incubated at 37°C for 1 h. The subsequent substrate coloring step was performed using TMB solution and incubated at 37°C for half an hour. The termination reaction was carried out using 0.05 mL sulfuric acid (2 M) per pore. The OD value at 450 nm of each sample was detected by the ELISA Microplate Reader.

### 2.13 Cell migration assay

LX‐2 cells were divided into Furin + TGF‐βgroup and NC + TGF‐βgroup in the cell migration assay. Cell transwell assay was used for evaluation of cell migration capacity in different treatment groups. LX‐2 cells were incubated with DMEM solution without fetal bovine serum (FBS) for 24 h before the Transwell assay. LX‐2 cells were resuspended in DMEM solution and added into the upper chamber of the transwell at a density of 5,000/200 μL. 24 h later, transwells were put into new 24‐well plates and cells on the transwell membrane were stained using crystal violet solution and washed with PBS three times. Stained cells were observed under a microscope and counted in different magnification views.

### 2.14 CCK‐8 cell viability assay

Cell Counting Kit‐8 (Dojindo, Japan) was used in this study for cell viability detection. LX‐2 cells were seeded into 96‐well plates and cultured at 37°C, 5% CO_2_ condition overnight until attached growth. Then the LX‐2 cells in different treatment groups were added 10 μL CCK‐8 reagent per well and incubated at 37°C condition for at least 1 h. Afterwards, 96‐well plates were put into the Microplate Reader, and absorbance values at 450 nm were detected and recorded detailedly.

### 2.15 Statistical analysis

The data was presented as mean ± standard deviation (SD), and statistical significance between two groups was analyzed using Student's t test. Besides, statistical significance among three groups was analyzed using the one‐way ANOVA method. *p* < 0.05 was considered statistically significant. Statistical analyses were performed by using SPSS Statistics 21 software (Release version 21.0.0.0, IBM Corp, Armonk, NY, USA).

## 3 RESULTS

### 3.1 Furin is a positive expression in cirrhotic liver tissues and is up‐regulated during HSCs activation

**FIGURE 1:**
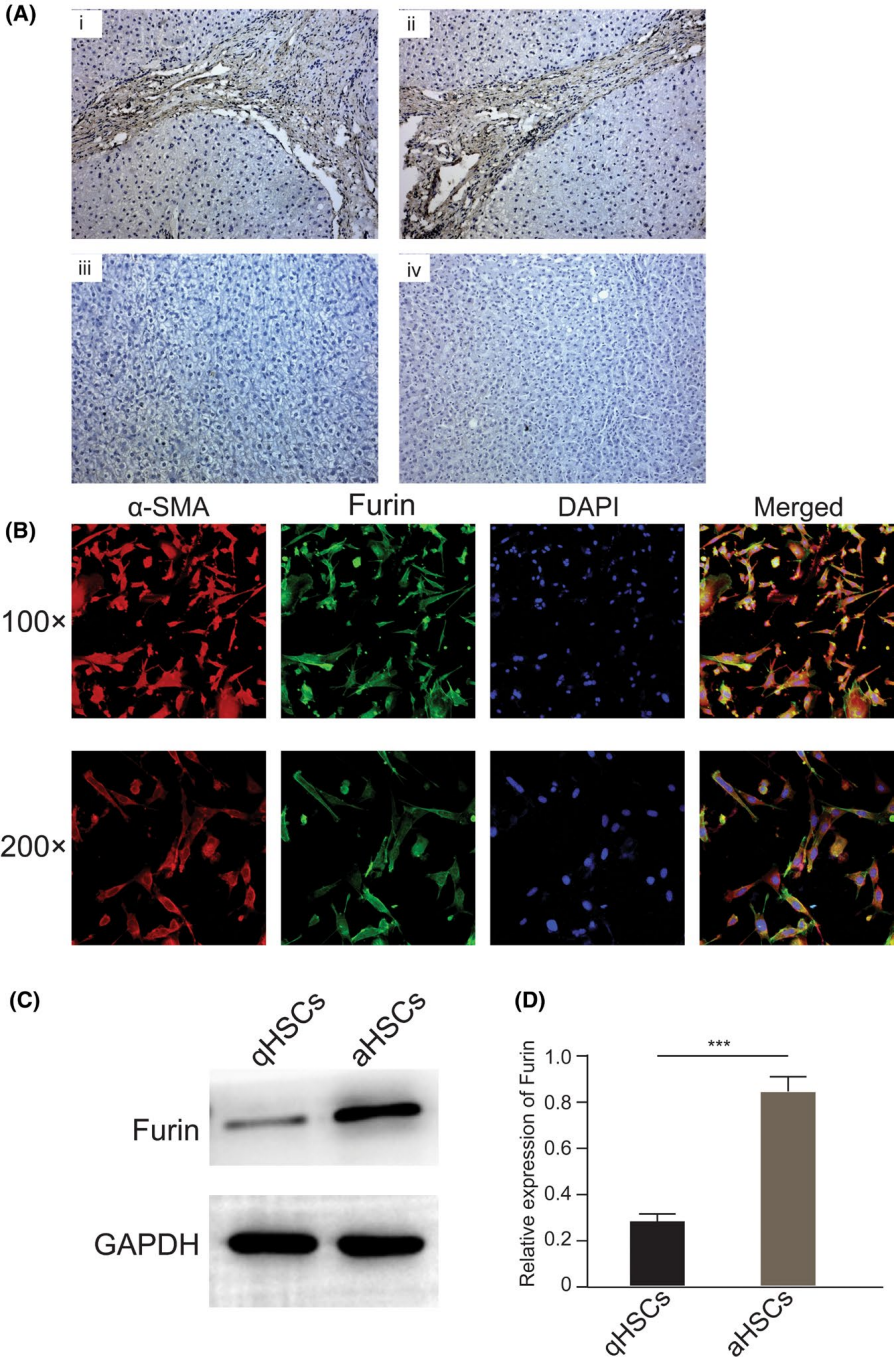
Furin expression is up‐regulated both in cirrhotic liver tissues and activated HSCs. (A) (i) and (ii) Immunohistochemical staining of Furin in cirrhotic liver tissues; (iii) and (iv) Immunohistochemical staining of Furin in normal liver tissues; (B) Immunofluorescence images of Furin (green), α‐SMA (red) and DAPI (blue) in mouse primary HSCs that were cultured in vitro until activated. (C) Western blot results showed that relative expression of Furin in activated HSC (aHSCs) was higher than that in quiescent HSCs (qHSCs). (D) Quantitified result of (C) by using ImageJ software.

Furin expression in human liver tissues was also detected using IHC staining, and the results indicated that Furin was obviously positively expressed in human cirrhotic liver tissues compared with normal liver tissues (Figure 1A). We isolated primary HSCs from healthy C57 mice aimed to identify aberrant Furin expression during HSC activation. The immunofluorescence staining results showed that Furin expression was significantly up‐regulated during the HSC activation process in parallel with the HSC activation marker α‐SMA expression (Figure 1B). Moreover, western blot results revealed that Furin expression in activated HSCs (aHSCs) was quite higher than that in quiescent HSCs (qHSCs) (Figure 1C,D). These results suggested that Furin might play an important role in regulating HSC activation.

### 3.2 Furin inhibits HSCs activation, proliferation and migration

**FIGURE 2:**
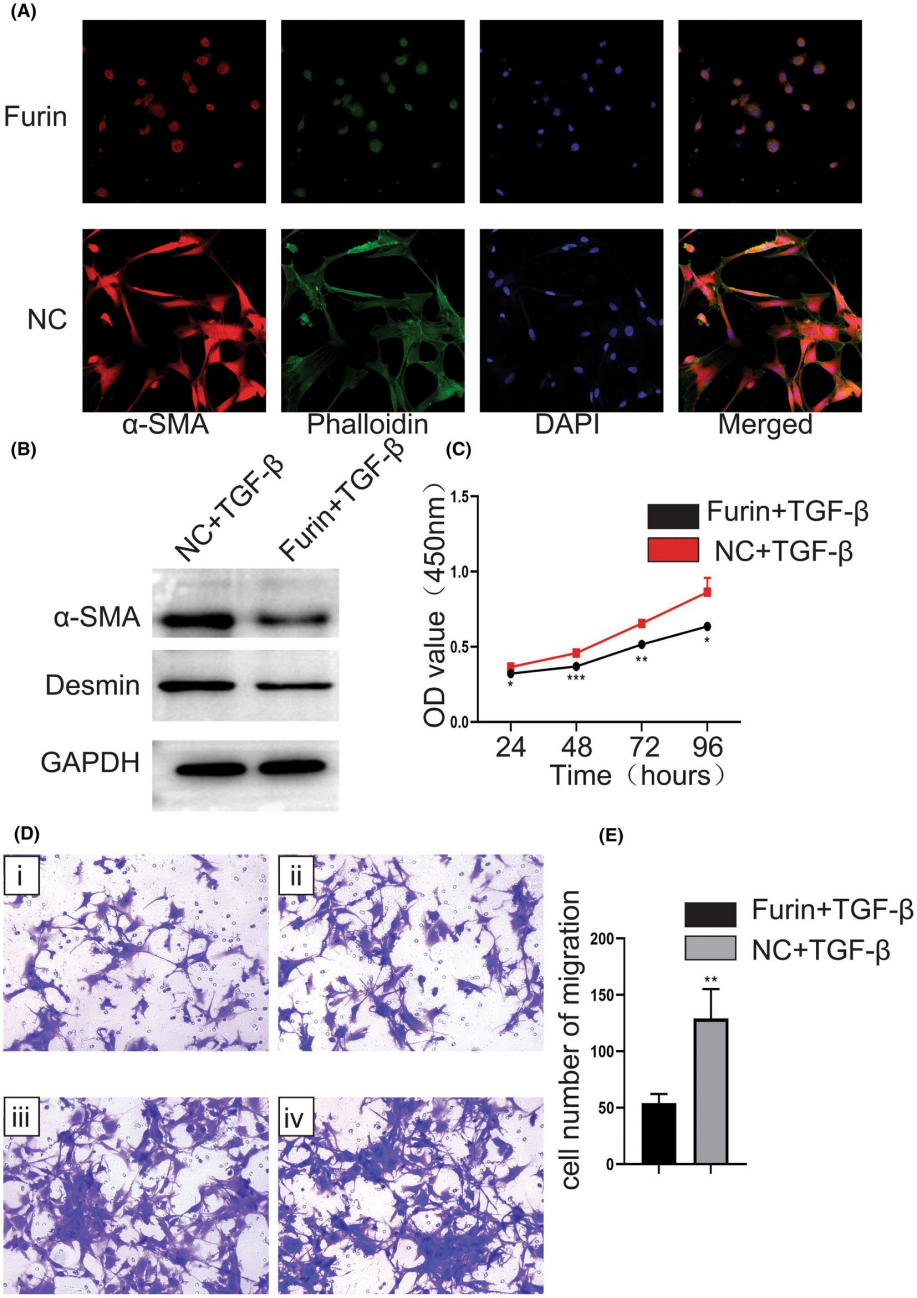
Furin inhibits HSCs activation and regulates migration and proliferation of the LX‐2 cells in vitro. (A) Immunofluorescence microscope images of primary mouse HSCs under different treatments: Phalloidin (green), α‐SMA (red) and DAPI (blue). Primary HSC activation was inhibited by Furin treatment. (B) Western blot results showed that HSC activation markers α‐SMA and Desmin were down‐regulated under Furin treatment. (C) CCK‐8 assay showed that the proliferation of LX‐2 cells was inhibited by Furin treatment. (D) Transwell experiment showed that the migration capacity of LX‐2 cells was inhibited by Furin treatment (i, ii: Furin treated; iii, iv: NC). (E) Column chart of (D) result.

In order to investigate the effect of Furin on primary HSCs activation, we carried out functional experiments in vitro. Compared with the control group, primary mice HSCs activation in the Furin treatment group was obviously inhibited paralleled with the decrease of fibrotic markers expression (Figure 2A). Western Blot results showed that the expression of HSCs activation markers α‐SMA and Desmin in the Furin treatment group was significantly lower than that in NC group cells (Figure 2B). We also performed a CCK‐8 assay in LX‐2 cells and revealed that Furin overexpression led to inhibition of proliferation in LX‐2 cells (Figure 2C). The transwell results indicated that Furin over‐expression caused inhibition of LX‐2 cells migration in vitro (Figure 2D,E).

### 3.3 Furin promotes PINK1/Parkin mediated HSCs mitophagy

**FIGURE 3:**
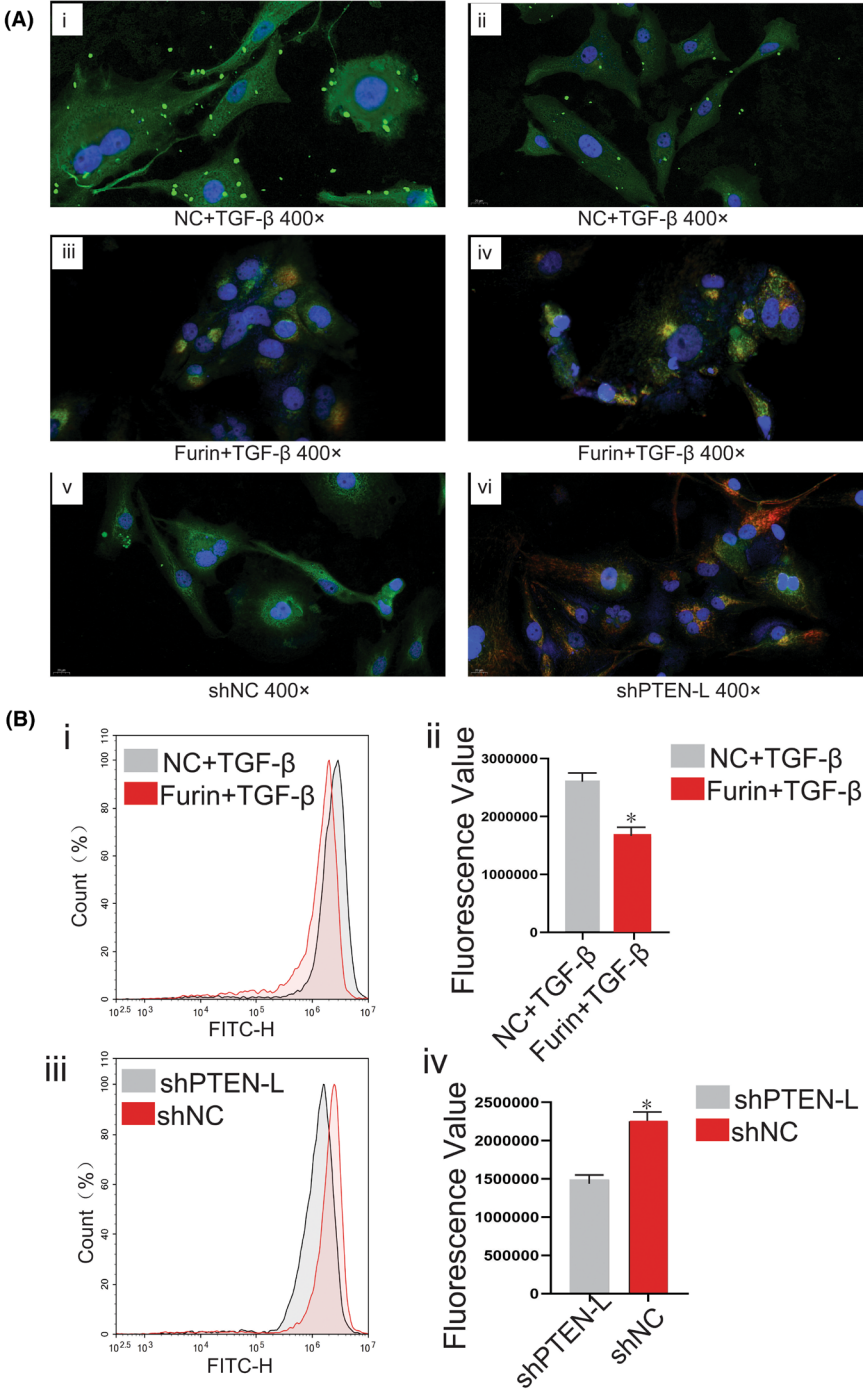
Furin promoted HSCs mitophagy and caused decrease of MMP. (A) Fluorescence microscope images of HSCs under different treatments: OPTN (green), Tom20 (red), DAPI (blue), OPTN and Tom20 colocalization (yellow). (B) Flow cytometry results of HSCs under different treatments; The cell mitochondrial membrane potential (MMP) was detected by using JC‐1 staining method.

We also detected molecular cellular colocalization between Tom20 and OPTN (optineurin, autophagy receptor protein) in different treatment groups to further reveal the regulation of mitophagy by Furin. The immunofluorescence assay results showed that Tom20 and OPTN cellular colocalization in HSCs was significantly increased in the TGF‐β plus Furin (TGF‐β + Furin) treatment group compared with TGF‐βalone treatment (Figure 3Ai–iv). Interestingly, PTEN‐L knockout also led to the increase of Tom20 and OPTN colocalization in primary HSCs compared with the control group (Figure 3Av–vi). Cellular colocalization of Tom20 and OPTN was considered an important sign of HSCs mitophagy. We detected HSCs mitochondrial membrane potential changes under different treatments in order to investigate the role of Furin in mitophagy regulation. Our results showed that TGF‐β plus Furin (TGF‐β + Furin) treatment led to a significant decline of mitochondrial membrane potential compared with TGF‐βalone treatment (Figure 3Bi,ii). Meanwhile, PTEN‐L knockdown also caused a significant decrease of mitochondrial membrane potential compared with the control group HSCs (Figure 3Biii,iv).

**FIGURE 4:**
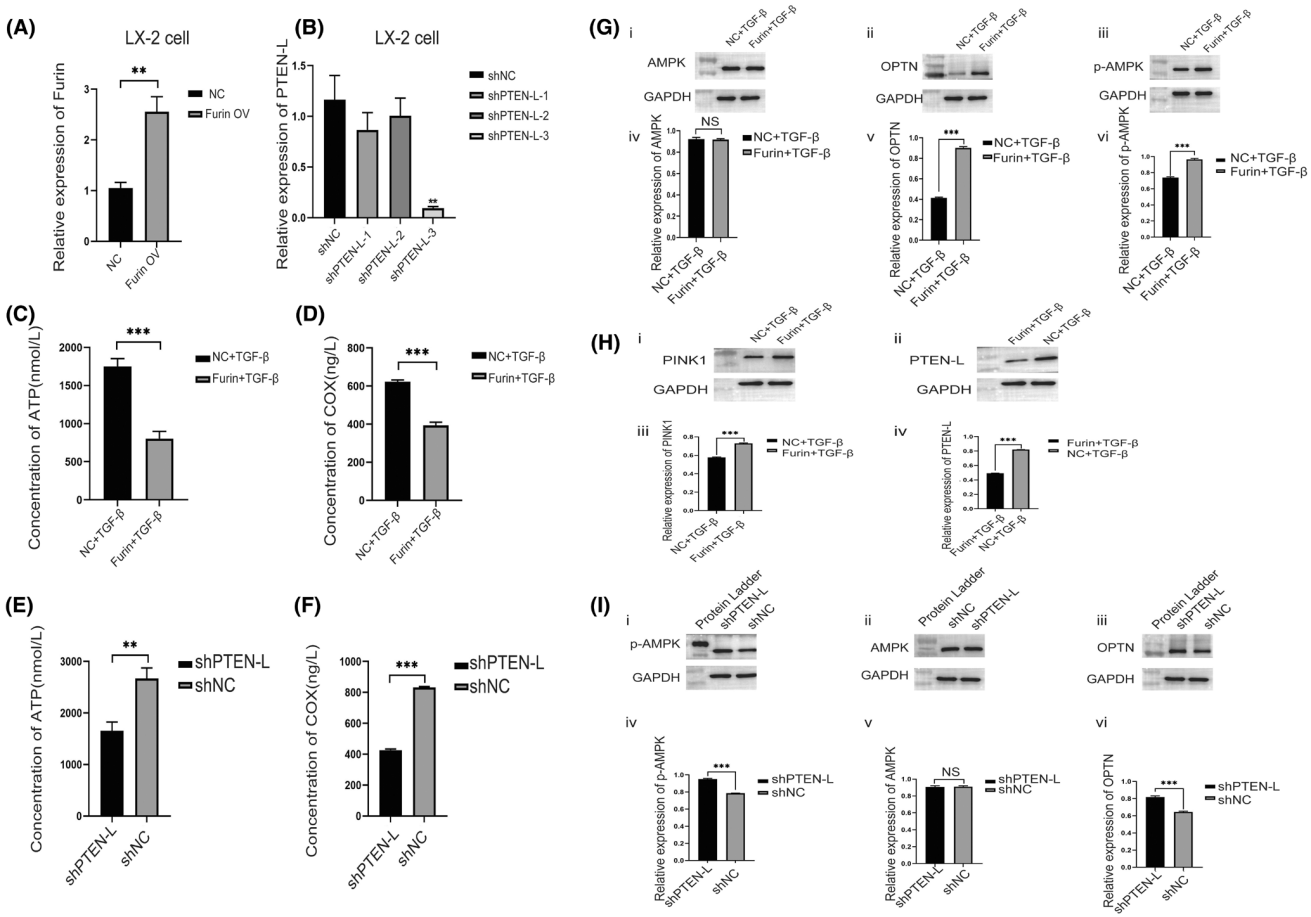
The role of Furin in regulating ATP and COX expression of HSCs. (A) LX‐2 cells were transfected with lentivirus to achieve Furin over‐expression; (B) PTEN‐L expression in LX‐2 cells was knocked down by the shRNA transfection method. (C) Detection of ATP in HSCs under Furin and NC treatment; (D) Detection of COX in HSCs under Furin and NC treatment; (E) Detection of ATP in HSCs under PTEN‐L shRNA transfection; (F) Detection of COX in HSCs under PTEN‐L shRNA transfection. The role of Furin and PTEN‐L in regulating HSC mitophagy related signal molecule markers. (G) Western blot results showed the changes in AMPK (i, iv), OPTN (ii, v), p‐AMPK (iii, vi) expression caused by Furin treatment; (H) Western blot results showed the changes in PINK1 (i, iii), PTEN‐L (ii, iv) expression caused by Furin treatment; (I) Western blot results showed the changes in p‐AMPK (i, iv), AMPK (ii, v), OPTN (iii, vi) expression caused by PTEN‐L knockdown treatment.

The production of ATP can be considered an important indicator of mitochondrial function. Herein, we carried out Furin overexpression and PTEN‐L knockdown assays in LX‐2 cells to evaluate the impact of Furin on ATP production in HSCs (Figure 4A,B). ATP (Adenosine triphosphate) concentration detection results indicated that ATP was significantly decreased in the TGF‐β plus Furin (TGF‐β + Furin) treatment group compared with the TGF‐βalone treatment (Figure 4C). The Cox (Cytochrome oxidase) was also considered an important indicator of mitochondrial function assessment. The Cox detection both in the Furin treatment group (TGF‐β + Furin) and the TGF‐βalone treatment group showed similar results to ATP detection (Figure 4D). Meanwhile, our results showed that PTEN‐L knockdown also caused the decrease of ATP and Cox concentration compared with the control group in HSCs (Figure 4E,F).

We performed several experiments to further reveal the impact of Furin on HSC mitophagy. Our results showed that OPTN and phospho‐AMPK (p‐AMPK) expression in the Furin treatment group (TGF‐β + Furin) were obviously higher than that in the NC group (Figure 4Gi–iii,iv–vi). Besides, western blot results also showed that PINK1 expression (the PINK1 full‐length, from https://pubmed.ncbi.nlm.nih.gov/24121706/) in the Furin treatment group (TGF‐β + Furin) was higher than that in the NC group (Figure 4Hi,iii). Moreover, western blot results indicated that the expression of PTEN‐L was lower in the Furin treatment group (TGF‐β + Furin) compared with the NC group, which may be attributed to molecular shearing by Furin (Figure 4Hii,iv). Interestingly, PTEN‐L knockdown in HSCs also led to up‐regulated expression of p‐AMPK and OPTN compared with the NC group (Figure 4Ii–vi).

These data indicated that Furin promotes PINK1/Parkin‐mediated HSCs mitophagy in vitro.

### 3.4 Furin alleviates liver fibrosis progression in vivo

**FIGURE 5:**
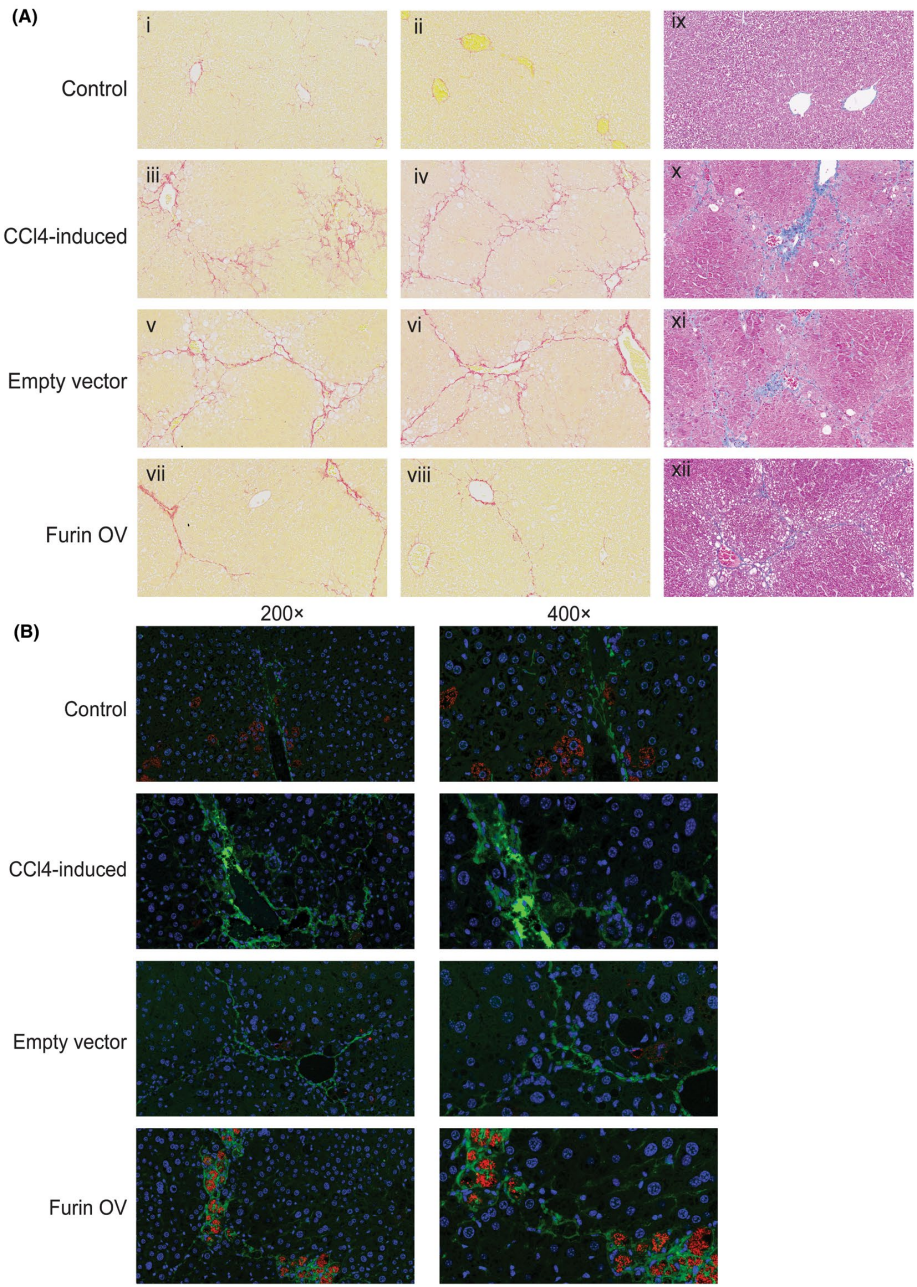
Furin alleviated mice liver fibrosis in vivo. (A) Sirius Red staining images of liver tissues in control (i, ii), CCl4‐induced (iii, iv), CCl4‐induced with lentivirus empty nonsense vector transfection (v, vi), CCl4‐induced with lentivirus Furin overexpression groups (vii, viii) under 200× amplification. Masson's trichrome staining images of liver tissues in control (ix), CCl4‐induced (x), CCl4‐induced with lentivirus empty nonsense vector transfection (xi), CCl4‐induced with lentivirus Furin overexpression groups (xii) under 200× amplification. Furin promoted HSCs mitophagy in vivo. (B) Immunofluorescence microscope images of mice liver tissues under different treatments as described in Figure 6. The Immunofluorescence images including OPTN staining (green), HSP60 staining (red), DAPI (blue) under 200×, 400× amplification respectively.

Based on the above results, we performed a chemical‐induced fibrosis mouse model to explore the role of Furin in liver fibrosis progression. The C57 mice were divided into four groups as follows: control group, model group, empty vector transfection group, and Furin overexpression (OV) group. We chose the CCl4‐induced method for fibrosis model construction in our study, and the Masson's/Sirius Red stain results showed that the severity of mice liver fibrosis in the Furin OV group was quite alleviated compared with the empty vector model group (Figure 5Ai–viii for Sirius Red; ix‐xii for Masson stain).

The immunofluorescence assay results showed that the tissue colocalization of HSP60 (Red) and OPTN (green) in HSCs was significantly increased in the Furin OV group mice liver compared with the empty vector model group and the model group (Figure 5B).

**FIGURE 6:**
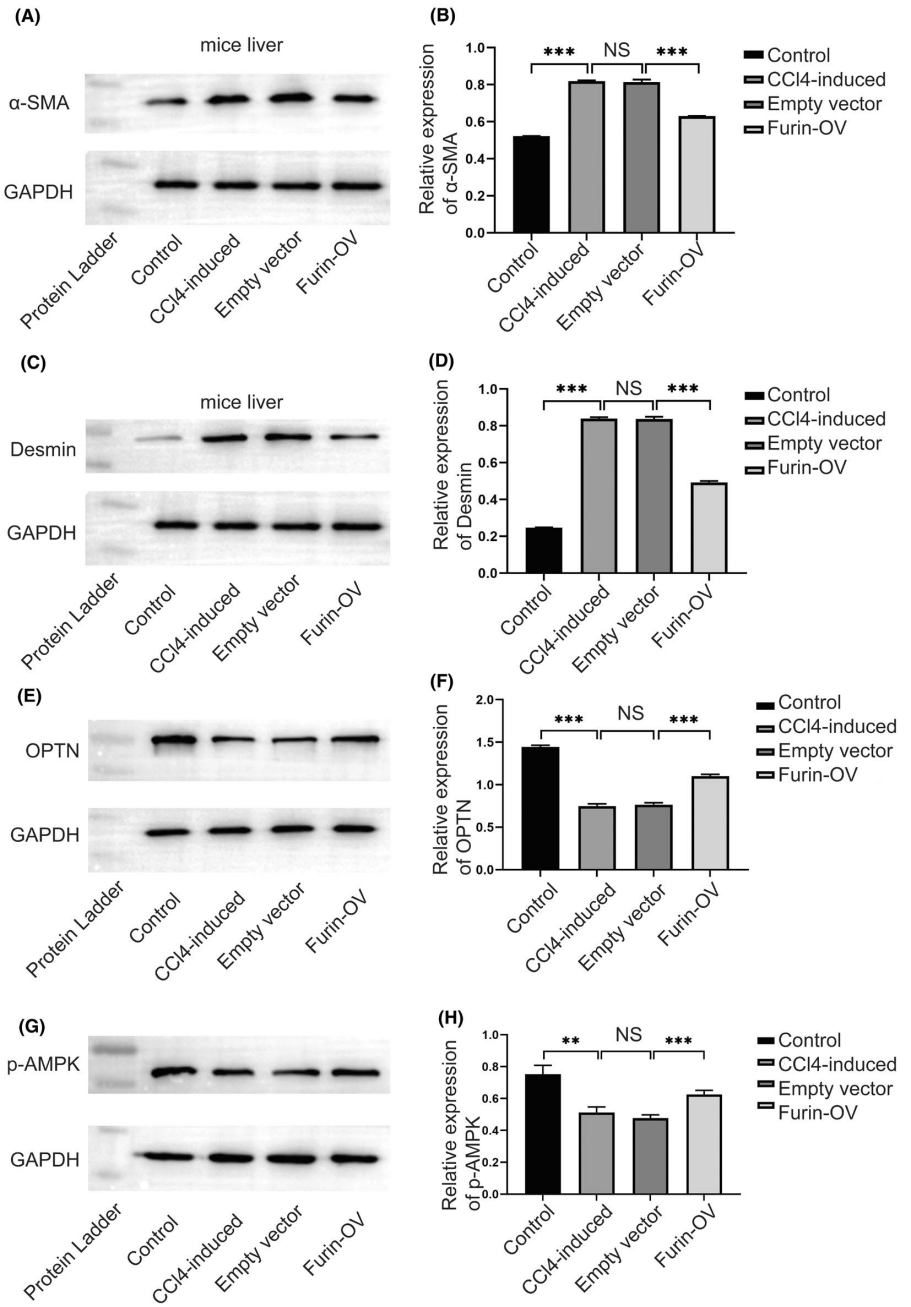
Furin regulated the expression of HSCs activation markers and mitophagy‐related molecule markers. (A, B) Western blot results showed the relative expression of α‐SMA of mouse liver tissues in different groups; (C, D) Western blot results showed the relative expression of Desmin of mice liver tissues in different groups; (E, F) Western blot results showed the relative expression of OPTN of mice liver tissues in different groups; (G, H) Western blot results showed the relative expression of p‐AMPK of mice liver tissues in different groups.

Western blot results showed that α‐SMA and Desmin expression in mice liver tissues of the Furin OV group were lower than that in both the empty vector model group and the CCl4‐induced model group (Figure 6A–D). However, the expression of OPTN in the Furin OV group was dramatically higher than that in the empty vector and model groups mice liver (Figure 6E,F). The expression of p‐AMPK in the Furin OV group mice liver was higher than that in both the empty vector model group and the model group (Figure 6G,H). Therefore, these results strongly proved that Furin can alleviate liver fibrosis progression and promote mitophagy in mice liver.

### 3.5 Furin can directly interact with PTEN‐L

**FIGURE 7:**
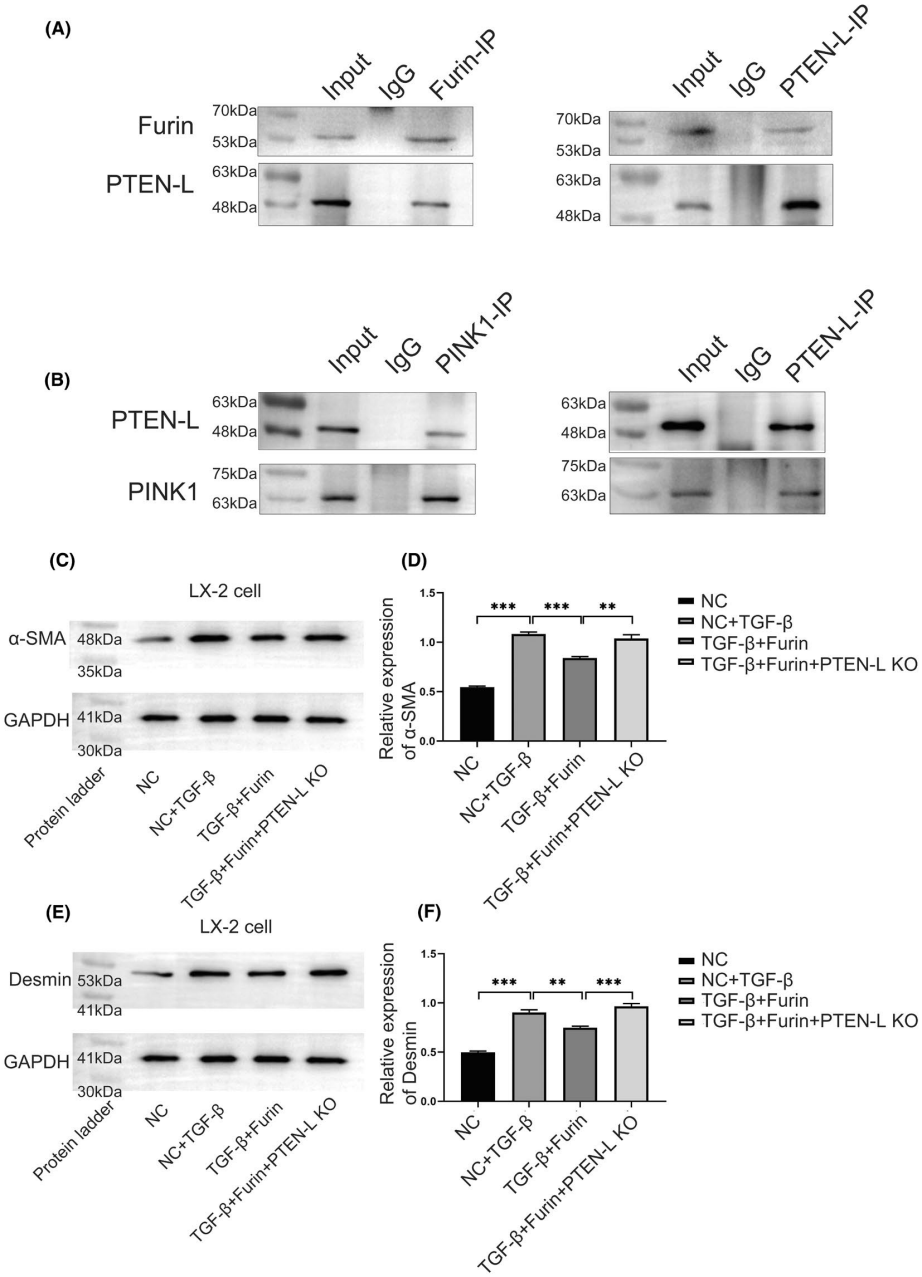
Furin can directly interact with PTEN‐L and inhibit HSC activation in a PTEN‐L‐dependent manner. (A) CO‐IP results showed that Furin can bind with PTEN‐L as a bait protein in LX‐2 cells; Besides, PTEN‐L can also bind with Furin directly in LX‐2 cells as a bait protein. (B) CO‐IP results showed that PINK1 can bind with PTEN‐L as a bait protein in LX‐2 cells; Besides, PTEN‐L can also bind with PINK1 directly in LX‐2 cells as a bait protein. (C, D) Western blot results revealed the changes in α‐SMA expression in LX‐2 cells caused by Furin treatment, and this inhibitory effect can be reversed by PTEN‐L knockout. (E, F) Western blot results revealed the changes in Desmin expression in LX‐2 cells caused by Furin treatment, and this inhibitory effect can be reversed by PTEN‐L knockout.

Accumulated reports indicated that PTEN protein can be sheared by Furin, causing significant changes in molecular function. In this study, we focused on the regulation of HSCs mitophagy mediated by the PTEN‐L/ PINK1/Parkin pathway. Therefore, it is important to confirm the direct interaction between Furin and PTEN‐L in HSCs. The IP (Immunoprecipitation) results showed that PTEN‐L expression can be detected from the captured protein complex precipitated by Furin IP‐specific antibodies (Figure 7A). Besides, we also successfully detected Furin expression from the captured protein complex precipitated by PTEN‐L IP antibodies (Figure 7A). The IP assay in LX‐2 cells also revealed the interaction between PTEN‐L and PINK1 (Figure 7B). These bidirectional verification results strongly supported the direct interaction between Furin and PTEN‐L in HSCs.

### 3.6 Furin promotes HSCs mitophagy via a PTEN‐L‐dependent manner

In order to further investigate the mechanisms of how Furin regulates mitophagy through the PTEN‐L/PINK1/Parkin pathway, we performed PTEN‐L knockout and recovery experiments.

We divided LX‐2 cells into four groups as follows, NC group, NC + TGF‐β treated group, TGF‐β + Furin OV group, and TGF‐β + Furin OV + PTEN‐L KO (knockdown) group. The western blot results showed that the expression of the HSCs activation marker α‐SMA in NC + TGF‐βgroup was significantly higher than that in NC group (Figure 7C,D). Interestingly, the expression of α‐SMA in the TGF‐β + Furin OV group was lower than that in NC + TGF‐βgroup (Figure 7C,D). However, expression of α‐SMA in TGF‐β + Furin OV + PTEN‐L KO group was significantly higher than that in TGF‐β + Furin OV group (Figure 7C,D). These results indicated that the inhibitory effect of Furin on α‐SMA expression can be partly reversed by PTEN‐L knockdown treatment. The expression of desmin in different groups detected by western blot was similar with α‐SMA (Figure 7E,F).

These data strongly suggested that Furin played an important role in regulating HSC activation and its inhibitory role mainly depends on the interaction with PTEN‐L.

## 4 DISCUSSION

Liver fibrosis is caused by various chronic diseases, including chronic HBV infection, alcohol intake, autoimmune liver diseases, metabolic liver diseases, and non‐alcoholic fatty liver disease.^3^ Liver fibrosis brings a great burden to the public health system in our country and will eventually proceed to liver cirrhosis.^9^ The cognition of the pathogenesis of liver fibrosis is limited, and effective treatment remains unclear.

It is widely accepted that HSCs activation is the key link of liver fibrosis.^10^, ^11^ Accumulated studies about liver fibrogenesis mainly focused on exploring regulatory mechanisms of HSCs activation.^5^, ^12^ Therefore, further investigation of the new mechanisms of HSCs activation can reveal potential therapeutic approaches against liver fibrosis and bring new insights for the pathogenesis of liver fibrosis. In this study, we revealed that Furin could inhibit HSCs activation and liver fibrosis progression in mice. Besides, our results also showed that HSCs mitophagy can be promoted by Furin treatment in vitro. These encouraging results revealed the important role of Furin in liver fibrogenesis and drove us to explore its related molecular mechanisms.

Mitophagy is an important mitochondrial quality control mechanism that plays an important role in maintaining the homeostasis of the intracellular microenvironment, which leads to the reduction of HSCs activation and alleviation of liver fibrogenesis.^13^, ^14^ Therefore, investigation of mitophagy in HSCs may provide new insights for understanding liver fibrogenesis. Mitophagy is responsible for the clearance of damaged mitochondria and can participate in the pathogenesis of metabolic syndrome, organ fibrosis, and tissue injury.^15^ Specifically, deregulation of mitophagy is also involved in the development of liver steatosis, hepatic fibrosis, viral hepatitis, and even liver cancer.^16^, ^17^ The correlation between mitophagy and HSCs activation was revealed by several previous studies. It was reported that PINK1 expression was down‐regulated and HSCs mitophagy was inhibited during HSCs activation.^18^ Previous studies also suggested that the inhibition of HSCs mitophagy caused progression of liver fibrosis in mice.^19^, ^20^, ^21^ Our results indicated that inhibition of mitophagy caused HSCs activation.^22^ Moreover, this study also showed that HSCs activation was inhibited by inducing mitophagy.^23^ In this study, we found that Furin promoted HSCs mitophagy, explaining its inhibitory role on HSCs activation and liver fibrosis. Therefore, this study aimed to provide a new mitophagy modulation‐related therapeutic target for treating and intervening in liver fibrosis and related chronic liver diseases.

The PINK1 (PTEN‐induced putative kinase1)‐dependent protein phosphorylation and Parkin E3 ubiquitin ligase‐mediated protein ubiquitination pathway are the most well‐studied mechanisms for regulating mitophagy.^18^ PINK1/Parkin‐dependent mitophagy was indicated to be necessary for mitochondrial uncoupler‐induced mitophagy and programmed mitophagy.^24^, ^25^, ^26^, ^27^, ^28^ PTEN‐L (PTEN‐long) was considered a novel protein phosphatase to inhibit PINK1‐Parkin mediated mitophagy.^6^ The Parkin E3 ubiquitin ligase can be inhibited by PTEN‐L.^6^ More importantly, it was reported that secreted PTEN‐L can be cleaved by Furin.^29^, ^30^ The cleaved fragments of PTEN‐L showed different or even opposite functions in regulating tumor progression.^7^, ^31^ In this study, we innovatively confirmed that Furin directly interacted with PTEN‐L in HSCs. Moreover, we also verified that the fibrosis‐suppressive role of Furin depended on PTEN‐L. In addition, N‐terminal and C‐terminal fragments of PTEN‐L cleaved by Furin may play different roles in mitophagy regulation. This scientific issue needs to be further investigated in subsequent studies.

Therefore, we can conclude that Furin interacts with PTEN‐L and causes enhancement of PINK1/Parkin mediated mitophagy in HSCs. Overall, we conclude that Furin inhibits HSC activation and liver fibrosis through regulating PTEN‐L/PINK1/Parkin mediated mitophagy.

## AUTHOR CONTRIBUTIONS

Ming‐ze Ma and Yu‐hua Zhu designed this research; Yan‐wei Song and Yu‐hua Zhu analyzed data; M. Ming‐ze Ma, Yan‐wei Song, and Yu‐hua Zhu performed research; Ming‐ze Ma and Yan‐wei Song wrote the paper; Yu‐hua Zhu contributed new reagents or analytic tools; and Yan‐wei Song and Ming‐ze Ma developed software necessary to perform and record experiments.

## FUNDING INFORMATION

Natural Science Foundation of Shandong Province, Grant/Award Number: ZR2020MH240; National Natural Science Foundation of China, Grant/Award Number: 82000579.

## CONFLICT OF INTEREST STATEMENT

All the authors declare no conflict of interest in this article.

## Data Availability

The data that support the findings of this study are available in the results of this article. All the original data of any additional information that support the findings of this study will be available on request from the corresponding author.
